# Closed Depressed Skull Fracture in Childhood Reduced with Suction Cup Vacuum Method: Case Report and a Systematic Literature Review

**DOI:** 10.7759/cureus.5205

**Published:** 2019-07-23

**Authors:** Matheus F Ballestero, Ricardo S De Oliveira

**Affiliations:** 1 Division of Neurosurgery, Department of Medicine, Federal University of São Carlos, Sao Carlos, BRA; 2 Division of Pediatric Neurosurgery, Department of Surgery and Anatomy, University of São Paulo, Ribeirao Preto, BRA

**Keywords:** traumatic brain injury, depressed skull fracture, breast pump, pediatric neurosurgery

## Abstract

Depressed skull fracture, also referred to as a “ping-pong ball” or “pond” fracture in neonates, is a common sign of traumatic brain injury in paediatric patients. The main causes of depressed skull fractures include labour and obstetric trauma in newborns and direct head trauma in older children. Skull depression rarely resolves spontaneously, and the surgical options include open cranioplasty and percutaneous microscrew elevation, among others. The use of negative pressure as a technique for fracture reduction has been described in a few papers. Here, we present a case-based review along with an illustrative case of depressed skull fracture reduced using the suction cup method via negative pressure. In addition, a Systematic Literature Review was performed to evaluate the safety of applying this procedure. The suction cup method is a feasible method to reduce depressed skull fracture in children, with minimum complications and no apparent long-term impairments.

## Introduction

Depressed skull fracture is a common symptom of paediatric patients with traumatic brain injury, and comprises up to 23% of all cranial fractures [1]. In newborns and infants, this type of lesion is also referred to as a “ping-pong ball” or “pond” fracture, which are “green-stick” fractures of the skull.

The main causes of depressed skull fractures include various perinatal factors in newborns (labour, obstetric trauma) and head trauma in older children [2].

Skull depression rarely resolves spontaneously, and this cosmetic defect can cause anxiety for parents, who are often more comfortable with an active intervention. The surgical options include open cranioplasty and percutaneous microscrew elevation [3].

The use of negative pressure for reduction was first described in the 1970s by Schrager, who successfully treated a skull depression using a vacuum pump [4]. Thereafter, only few papers have described this technique, along with related complications and success rate.

For this reason, we conducted a case-based review, and also present an illustrative case of depressed skull fracture reduced using the suction cup vacuum (SCV) method. Additionally, a literature review was performed to evaluate the safety of this procedure.

The typical clinical presentation of depressed skull fracture is a neonate deformity following vaginal delivery or even after difficult caesarean surgery. In older children, however, this fracture presents following direct head trauma.

The deformity is usually obvious on inspection, and skull radiographs show incomplete fracturing. When there is no cortical fracture accompanying the depression, the condition is named faulty fetal packing.

Although three-dimensional CT scans can provide more detailed information about the brain parenchyma, the radiation risks are not acceptable for all neonates or young children. Therefore, CT scans are reserved for deeper, extensive or more complex cases, or when neurological examination reveals alterations [5].

Some fractures can elevate spontaneously, there is some concern about the long-term cosmetic appearance and possible brain compression [6]. For this reason, fractures are traditionally treated with elevation procedures. The surgical techniques used to elevate the skull include the insertion of a periosteal elevator through a new burr hole at the margins of the fracture, with the depressed bone fragments forced upwards; insertion of the same elevator through the coronal or lambdoidal sutures, avoiding the need for burr holes [7]; insertion of a percutaneous screw or a hook through the center of the fracture and pulling upwards [3]; or performing a circumferential craniotomy around the fracture, remoulding and reinserting the bone [8]. Non-invasive reduction through applying digital pressure at the margins of the fracture has also been reported, which avoids further intervention [2].

The prognosis of depressed skull fracture in childhood with no associated brain damage is good, even when no reduction procedure is performed. Mandatory urgent surgery is indicated for cases where a cerebrospinal fluid leak is clearly recognised, a foreign body is detected, debridement of the local wound is necessary, wound infection, or when evacuation of a hematoma is necessary [9].

Depressed skull fracture has been claimed to be an independent risk factor for post-traumatic seizures; however, there is no consensus in the current literature for this association [10].

## Case presentation

**Figure 1:**
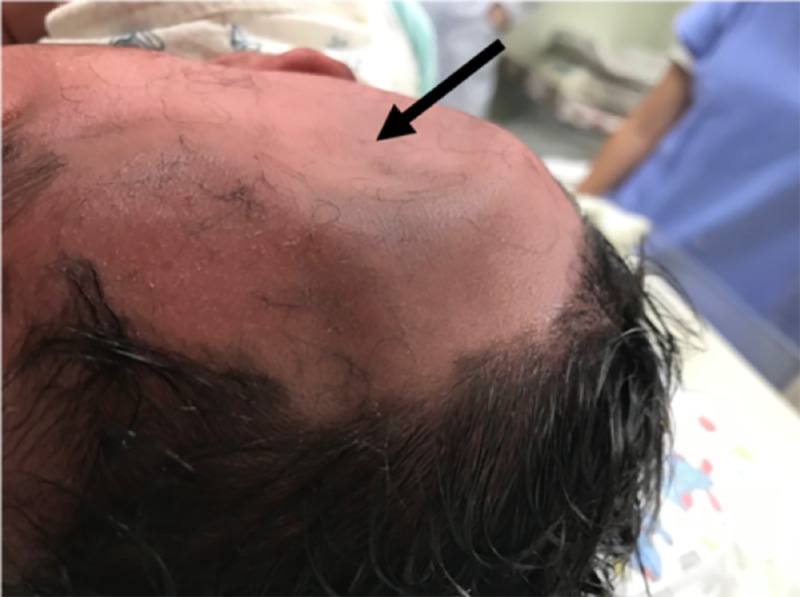
Illustrative case – Lateral head photograph showing the depressed skull fracture (black arrow).

A male neonate delivered by caesarean presented traumatism, identified as a right frontotemporal depressed skull fracture. The child weighed 4420 g, and was born after 37 weeks and four days by caesarean. The fracture was 4 cm in diameter by 1.5 cm in depth (Figure 1).

**Figure 2:**
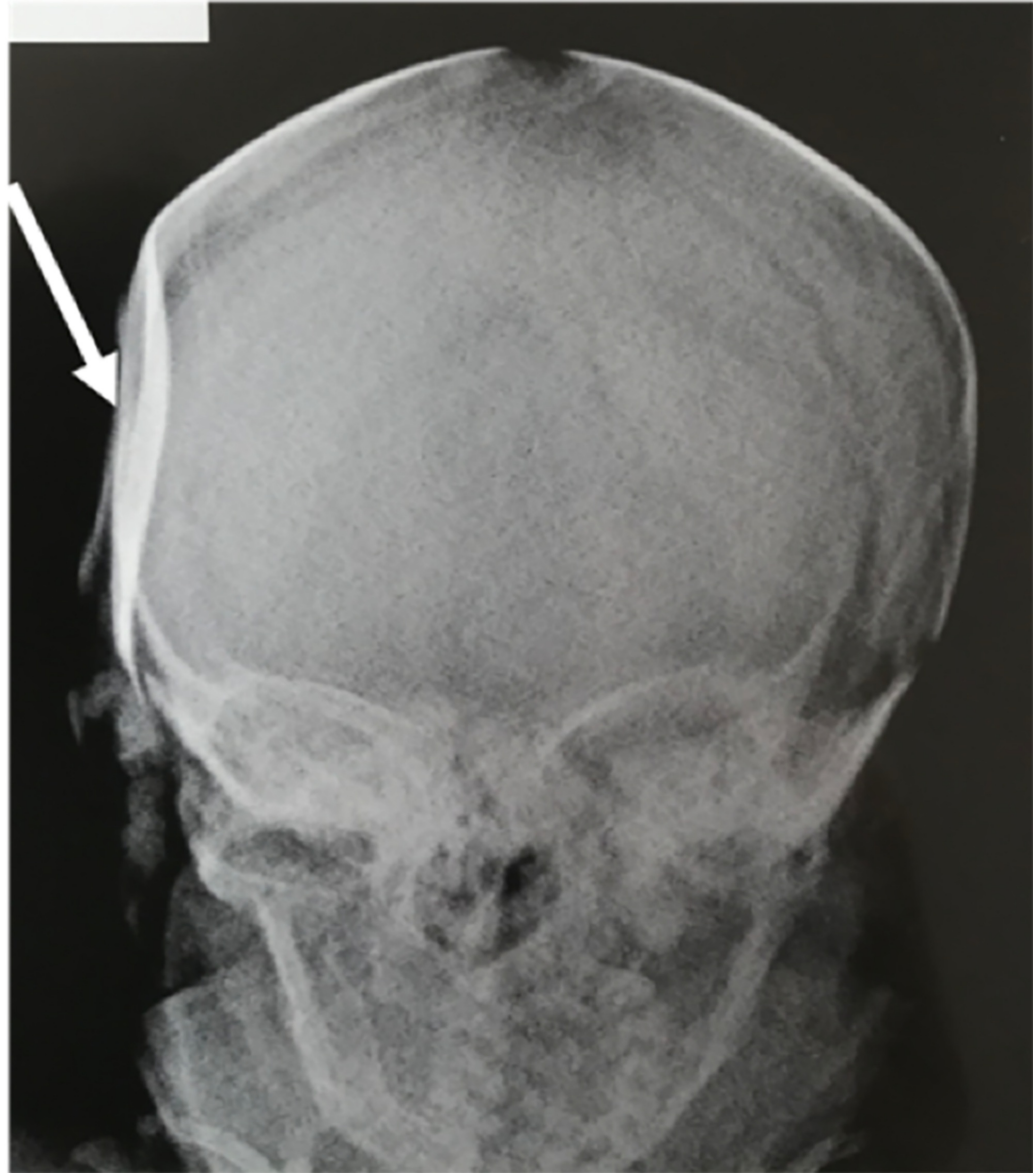
Illustrative case – Anteroposterior X-ray showing the bone deformity (white arrow).

Neurological examination and transfontanelle ultrasonography were unremarkable, but the deformity was visible by cranial X-ray (Figure 2).

After 72 h of observation, formal consent was obtained from the parents and the SCV method was applied to elevate the skull depression.

**Figure 3:**
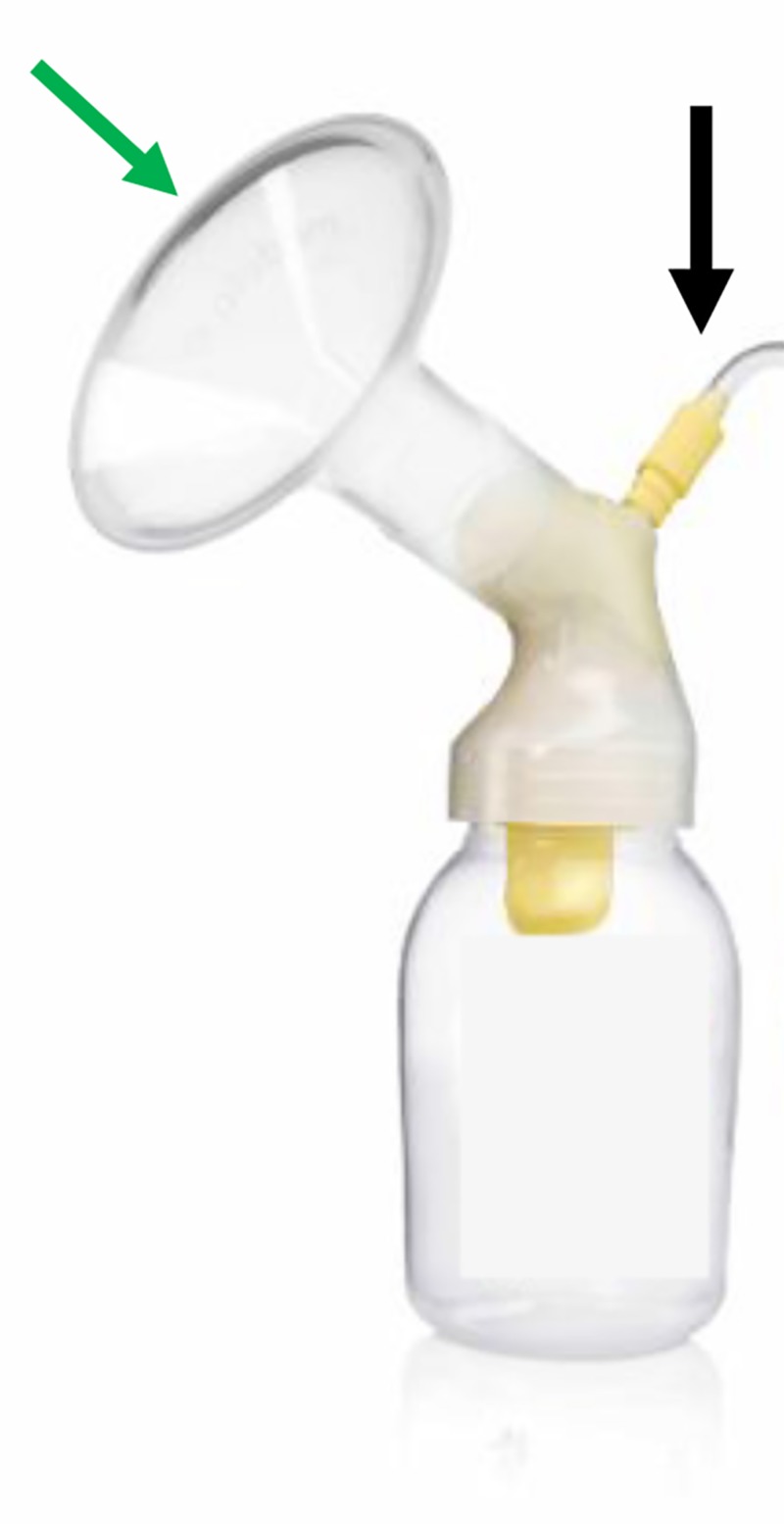
The breast milk extractor showing the vacuum connection (black arrow) and the surface used to create vacuum (green arrow).

A typical breast milk extractor (Figure 3) attached to hospital vacuum system was used for SVC. Elevation of the fracture was performed by placing the central part of the extractor over the central part of the defect after spreading solid vaseline over the defect to avoid air leakage. A progressive vacuum was applied while observing elevation through the breast milk extractor until the depression elevated and the sound of bone cracking could be heard.

**Figure 4:**
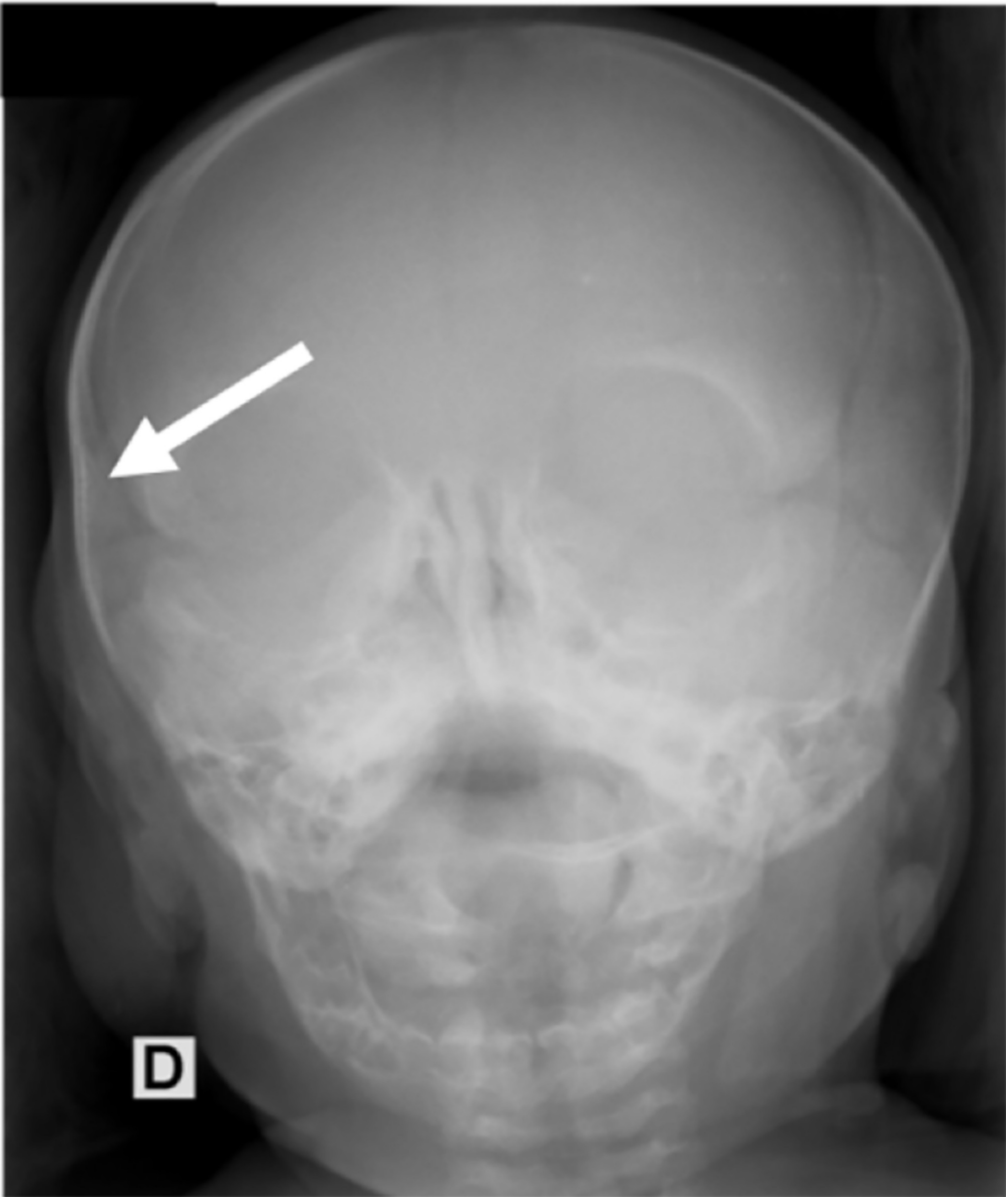
Illustrative case – Post-reduction anteroposterior X-ray showing no bone deformity (white arrow).

After the procedure, transfontanelle ultrasound was performed to confirm the absence of intracranial injury as a result of the procedure, and another cranium X-ray was performed to confirm that correct fracture reduction had occurred (Figure 4). The neonate was observed for 24 h before being discharged. At two months follow-up, the child presented normal development with a symmetric skull shape.

## Discussion

Systematic literature review

A literature search was performed to identify reports of the use of SCV to reduce skull fractures in children. Searches of the PubMed and Ovid MEDLINE databases were performed for relevant articles published until March 2019. Appropriate keywords and MeSH terms were used to identify all studies, including: “neonate”, “newborn”, “depressed skull fracture”, “vacuum extractor”, and “breast pump.” The reference lists of papers were also manually searched to identify additional data sources.

The literature review included eight papers with a total of 35 children submitted to skull fracture reduction through SCV (Table 1). The mean age ranged from a few hours to 18.5 months (mean 6.9 months). The sample contained more males (62.5%) than females (37.5%). The mechanism of trauma was peripartum trauma in 47.6%, falls in 19%, impact from falling objects in 14.3%, and road traffic accident in 14.3%.

**Table 1:** Summary of SCV technique used to reduce skull fracture in children published in the medical literature. SCV: Suction cup vacuum; CPH: Cephalohematoma; F: Female; FO: Falling object; FT: Frontal; FTTP: Frontotemporal; L: Left; M: Male; NI: No information; PT: Peripartum trauma; R: Right; RTA: Road traffic accident; TP: Temporal.

Study	Patients	Mean Age (mos)	Gender	Mechanism	Location	Side	Diameter (cm)	Success	Complications	Follow-up (mos)	Long-term impairment
Schrager, 1970 [4]	1	0	100% F	100% PT	100% PT	100% L	6.0	100%	None	12	None
Van Enk, 1972 [11]	1	5.55	100% M	100% Fall	100% PT	100% L	4.5	100%	None	18	None
Saunders et al., 1979 [12]	3	Neonate	100% M	100% PT	66.7% FT / 33.3% PT	66.6% L / 33.3% R	3.5	100%	None	NI	NI
Paul and Fahner, 1992 [13]	1	18	NI	100% Trauma	100% PT	100% R	NI	100%	None	NI	None
Pollak et al., 1999 [14]	1	Neonate	NI	100% PT	100% Parietal	100% L	4	100%	None	NI	NI
Hung et al., 2005 [15]	14	4.5	57.1% M / 42.9% F	NI	50% PT / 28.6% TP / 14.3% FT / 7.1% FTTP	57.1 R / 42.9 L	3.8	92.9%	None	24	None
Djientcheu et al., 2006 [16]	8	18.4	62.5% M / 37.5% F	37.5% Fall / 25% RTA / 37.5 FO	100% PT	50% R / 25% L / 25% NI	NI	100%	12.5% CPH	3	None
Kim et al., 2007 [17]	1	3	100% M	100% RTA	100% PT	100% R	NI	100%	None	NI	None
Mastrapa et al., 2007 [18]	5	1	60% M / 40% F	100% PT	NI	NI	NI	100%	None	18	None
Overall	35	6.9	62.5% M / 37.5% F	47.6% PT / 19% Fall / 14.3% FO / 14.3% RTA / 4.8% Other	70% PT / 13.3% FT/ 13.3% TP / 3.3% FTTP	42.9% Right / 37.1% Left / 20.0% NI	4.0	97.2%	2.9% CPH	16.6	None

Regarding the defect localisation, 70% were parietal, 13.3% frontal, 13.3% temporal and 3.3% frontoparietal. Of the fractures, 42.9% occurred on the right side and 37.1% on the left side, with no information on the location reported for the remaining 20% of children. The mean diameter of the depression was 4.0 cm.

The procedure was successful in 97.2% of cases, with complications observed in only one child (cephalohematoma). No long-term impairment was described over a mean follow-up period of 16.6 months.

Discussion

Although depressed skull fracture in neonates and children is unusual and spontaneous reduction may be possible, parents sometimes prefer more aggressive intervention due to the deforming aspect of such fractures.

Operations can carry complications such as surgical scarring and wound infection, along with anaesthetic challenges, which cannot be ignored in children of this age [19]. The length of stay in the hospital is another factor that may influence the choice of treatment. Use of the SCV method can reduce the length of hospital stay to less than 24 hours, and avoids unnecessary cannulation of peripheral or umbilical veins and the risks associated with these procedures [20].

Another important aspect is the social effect of the non-invasive procedure, as it prevents unnecessary parental stress and avoids interruption of breast-feeding and segregation from the mother.

Comparing our case with the literature, children were generally older in the review (mean 6.9 months). However, despite the variation in age, the predominant trauma mechanism was peripartum trauma (47.6%), showing a long delay between trauma and effective treatment.

The literature search did not reveal an age limit for SCV intervention. Djientcheu et al. were able to correct fracture in children from five months to 17 years (not a greenstick fracture) with 100% success, thereby expanding the therapeutic possibilities beyond single ping pong fractures [16].

## Conclusions

The suction cup method is a feasible method to reduce depressed skull fractures in children, and is associated with minimum complications and no apparent long-term impairments.

## References

[1] Harwood-Nash DC, Hendrick EB, Hudson AR (1971). The significance of skull fractures in children. A study of 1,187 patients. Radiology.

[2] Raynor R, Parsa M (1968). Nonsurgical elevation of depressed skull fracture in an infant. J Pediatr.

[3] Zalatimo O, Ranasinghe M, Dias M, Iantosca M (2012). Treatment of depressed skull fractures in neonates using percutaneous microscrew elevation. J Neurosurg Pediatr.

[4] Schrager GO (1970). Elevation of depressed skull fracture with a breast pump. J Pediatr.

[5] Cho SM, Kim HG, Yoon SH, Chang KH, Park MS, Park YH, Choi MS (2018). Reappraisal of neonatal greenstick skull fractures caused by birth injuries: comparison of 3-dimensional reconstructed computed tomography and simple skull radiographs. World Neurosurg.

[6] Axton JHM, Levy LF (1965). Congenital moulding depressions of the skull. Br Med J.

[7] Stein SC (2019). The evolution of modern treatment for depressed skull fractures. World Neurosurg.

[8] Rogers L (1953). Simple depressed fracture of the skull. Br Med J.

[9] Steinbok P, Flodmark O, Martens D, Germann ET (1987). Management of simple depressed skull fractures in children. J Neurosurg.

[10] Araki T, Yokota H, Morita A (2017). Pediatric traumatic brain injury: characteristic features, diagnosis, and management. Neurol Med Chir.

[11] Van Enk A (1972). Reduction of pond fracture. Br Med J.

[12] Saunders BS, Lazoritz S, McArtor RD, Marshall P, Bason WM (1979). Depressed skull fracture in the neonate. Report of three cases. J Neurosurg.

[13] Paul MA, Fahner T (1991). Closed depressed skull fracture in childhood reduced with suction cup method: case report. J Trauma.

[14] Pollak L, Raziel A, Ariely S, Schiffer J (1999). Revival of non-surgical management of neonatal depressed skull fractures. J Paediatr Child Health.

[15] Hung KL, Liao HT, Huang JS (2005). Rational management of simple depressed skull fractures in infants. J Neurosurg.

[16] de Paul Djientcheu V, Njamnshi AK, Ongolo-Zogo P, Ako S, Essomba A, Sosso MA (2006). Depressed skull fractures in children: treatment using an obstetrical vacuum extractor. Pediatr Neurosurg.

[17] Kim YJ, Lee SK, Cho MK, Kim YJ (2007). Elevation of depressed skull fracture with a cup of breast pump and a suction generator: a case report in technical aspects. J Korean Neurosurg Soc.

[18] Mastrapa TL, Fernandez LA, Alvarez MD, Storrs BB, Flores-Urueta A (2007). Depressed skull fracture in Ping Pong: elevation with Medeva extractor. Child's Nerv Syst.

[19] Escobar MA Jr, Caty MG (2016). Complications in neonatal surgery. Semin Pediatr Surg.

[20] Ramasethu J (2008). Complications of vascular catheters in the neonatal intensive care unit. Clin Perinatol.

